# Heparin Mimics Extracellular DNA in Binding to Cell Surface-Localized Proteins and Promoting *Staphylococcus aureus* Biofilm Formation

**DOI:** 10.1128/mSphere.00135-17

**Published:** 2017-06-21

**Authors:** Surabhi Mishra, Alexander R. Horswill

**Affiliations:** a Department of Microbiology, Carver College of Medicine, The University of Iowa, Iowa City, Iowa, USA; b Department of Veterans Affairs Iowa City Health Care System, Iowa City, Iowa, USA; University of Nebraska Medical Center

**Keywords:** heparin, *Staphylococcus aureus*, biofilm, eDNA, glycosaminoglycan, MRSA, extracellular DNA

## Abstract

*Staphylococcus aureus* is a leading cause of catheter-related bloodstream infections. Biofilms form on these implants and are held together by a matrix composed of proteins, polysaccharides, and extracellular DNA (eDNA). Heparin is a sulfated glycosaminoglycan that is routinely used in central venous catheters to prevent thrombosis, but it has been shown to stimulate* S. aureus* biofilm formation through an unknown mechanism. Data presented here reveal that heparin enhances biofilm capacity in many *S. aureus* and coagulase-negative staphylococcal strains, and it is incorporated into the USA300 methicillin-resistant *S. aureus* (MRSA) biofilm matrix. The *S*. *aureus* USA300 biofilms containing heparin are sensitive to proteinase K treatment, which suggests that proteins have an important structural role during heparin incorporation. Multiple heparin-binding proteins were identified by proteomics of the secreted and cell wall fractions. Proteins known to contribute to biofilm were identified, and some proteins were reported to have the ability to bind eDNA, such as the major autolysin (Atl) and the immunodominant surface protein B (IsaB). Mutants defective in IsaB showed a moderate decrease in biofilm capacity in the presence of heparin. Our findings suggested that heparin is substituting for eDNA during *S. aureus* biofilm development. To test this model, eDNA content was increased in biofilms through inactivation of nuclease activity, and the heparin enhancement effect was attenuated. Collectively, these data support the hypothesis that *S. aureus* can incorporate heparin into the matrix and enhance biofilm capacity by taking advantage of existing eDNA-binding proteins.

**IMPORTANCE** *Staphylococcus aureus* and coagulase-negative staphylococci (CoNS) are the leading causes of catheter implant infections. Identifying the factors that stimulate catheter infection and the mechanism involved is important for preventing such infections. Heparin, the main component of catheter lock solutions, has been shown previously to stimulate *S. aureus* biofilm formation through an unknown pathway. This work identifies multiple heparin-binding proteins in *S. aureus*, and it reveals a potential mechanism through which heparin enhances biofilm capacity. Understanding the details of the heparin enhancement effect could guide future use of appropriate lock solutions for catheter implants.

## Abstract (precis)

*Staphylococcus aureus* and coagulase-negative staphylococci (CoNS) are the leading causes of catheter implant infections. Identifying the factors that stimulate catheter infection and the mechanism involved is important for preventing such infections. Heparin, the main component of catheter lock solutions, has been shown previously to stimulate *S. aureus* biofilm formation through an unknown pathway. This work identifies multiple heparin-binding proteins in *S. aureus*, and it reveals a potential mechanism through which heparin enhances biofilm capacity. Understanding the details of the heparin enhancement effect could guide future use of appropriate lock solutions for catheter implants.

## INTRODUCTION

Inserting catheters to gain access to veins is common hospital practice. The challenge is that catheter implant material is readily colonized by skin commensal bacteria, which can persist on the catheter and gain access to the bloodstream. These infections are commonly referred to as catheter-related bloodstream infections (CRBSIs), and they can be a significant burden for both the patient and health care system. CRBSIs not only increase the length of hospital stay by 10 to 20 days, but they also increase the cost of patient care from $4,000 to $56,000 (1). Thus, there is a clear need to understand the mechanisms underlying CRBSI development and develop improved strategies for their prevention.

*Staphylococcus aureus* and *Staphylococcus epidermidis* are the two most commonly isolated bacteria from CRBSIs, followed by enterococci, aerobic Gram-negative bacilli, and yeast (2, 3). *S. aureus* is a human commensal and colonizes approximately 20% of the population, while 60% of the population are transient carriers (4). *S. aureus* implant infections are often characterized by the formation of biofilms, which are aggregates of bacteria encased in an extracellular matrix that protects them from host immune responses and antibiotic intervention (5). *S. aureus* can form biofilms as early as 24 h after catheter placement (6). The extent of biofilm formation inside catheters depends on the duration of catheterization and the properties of the fluids administered through them (7). Central venous catheters are often filled with heparin, a highly sulfated glycosaminoglycan (8), which is used as an anticoagulant to maintain catheter patency. A previous study by Shanks and coworkers showed that sodium heparin promotes *in vitro* biofilm formation with *S. aureus* (9). Recently, Ibberson et al. showed that heparin stimulates biofilm formation in methicillin-resistant *S. aureus* (MRSA) as well, but the mechanism remains unknown (10). Despite the fact that heparin has biofilm-enhancing properties and could be a risk factor for patients, it remains the lock solution of choice in most hospital settings.

Heparin is an anionic polysaccharide and interacts with a variety of proteins from bacteria as well as higher organisms (11). Physiologically, heparin is synthesized exclusively in mast cells (8). Mast cells are abundant at the boundaries between the environment and the host internal milieu and are involved in allergic and anaphylactic reactions. Therefore, it is not uncommon for bacteria present on skin and mucosa of the lungs to encounter heparin. In fact, Ronnberg et al. reported induction of multiple mast cell genes when mast cells were cocultured with *S. aureus*, although heparin biosynthesis genes were not observed (12). Many studies have focused on *in vitro* binding of proteins to heparin during fractionation, and therefore, these studies may not identify true physiological interactions. Nevertheless, heparin binding to proteins on the cell surfaces of several pathogenic microorganisms was found to be important, such as induction of protective immunity to *Neisseria meningitidis* (13), loss of virulence in *Candida albicans* (14), inhibition of the blood stage growth of *Plasmodium falciparum* (15), and invasion of gastrointestinal epithelial cells by *Cryptosporidium parvum* (16).

In the present study, the mechanism of heparin biofilm enhancement with *S. aureus* was investigated. Microscopy demonstrated that heparin was incorporated into the biofilm matrix, and protease treatment disintegrated heparin-containing biofilms. Biochemical and genetic approaches identified specific surface and matrix proteins that mediate assembly of heparin-containing biofilms, and some of the proteins identified are known extracellular DNA (eDNA)-binding proteins. Our findings suggest that these eDNA-binding proteins on the *S. aureus* surface associate with heparin and facilitate biofilm enhancement.

## RESULTS

### Heparin enhances biofilm formation in multiple strains of *S. aureus* and coagulase-negative staphylococci.

**FIG 1:**
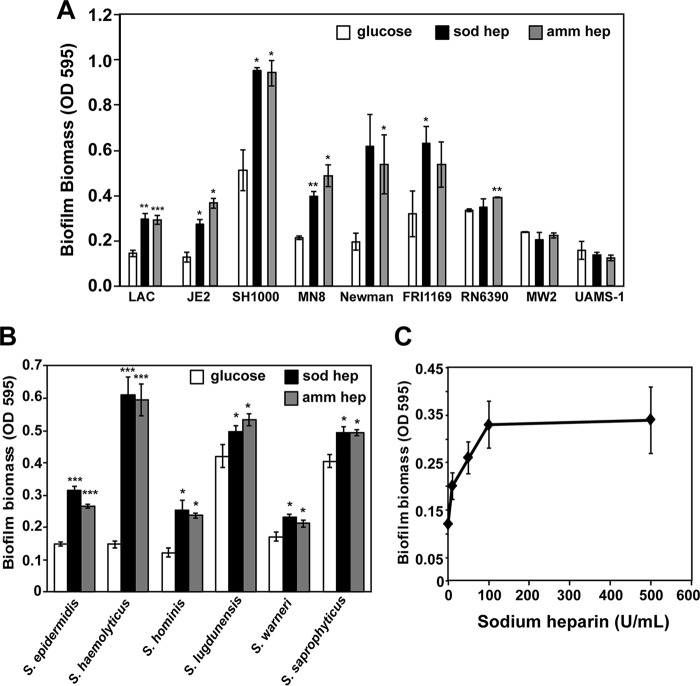
Heparin enhances *S. aureus* and coagulase-negative staphylococcus (CoNS) biofilm formation. (A and B) Microtiter biofilms of various *S. aureus* strains (A) or various CoNS species (B), grown in the presence or absence of sodium heparin (sod hep) or ammonium heparin (amm hep) as indicated. Values are the means ± standard deviations (error bars) from three independent experiments, with four technical replicates in each experiment. (C) Dose dependence of biofilm formation by strain LAC in the presence of increasing amounts of sodium heparin. A Student *t* test was performed, and statistical significance is indicated as follows: *, *P* < 0.05; **, *P* ≤ 0.005; ***, *P* ≤ 0.0005.

Shanks and coworkers reported that the addition of 1,000 U/ml of sodium heparin to the growth medium stimulated biofilm formation in *S. aureus* strain MZ100 and a few other related strains (9). To determine the prevalence of this phenomenon, the effect of heparin on biofilm formation was tested in various clinical isolates of *S. aureus*, including both methicillin-susceptible *S. aureus* (MSSA) and MRSA strains. A total of 9 strains of *S. aureus* were analyzed for biofilm formation in the presence of 100 U/ml of sodium heparin or ammonium heparin in an *in vitro* biofilm assay (17). A dose of 100 U/ml is a clinically relevant dose of sodium heparin and was used in all further experiments in this work unless mentioned otherwise (18). Ammonium heparin chemically resembles sodium heparin, and it was included in most of the experiments to rule out the effect of sodium addition alone. In biofilm microtiter assays, heparin stimulated increase in biofilm biomass for seven out of nine strains (Fig. 1A), and the phenomenon was spread across different strain lineages. Strains showing heparin-mediated increase in the assay also showed pronounced aggregation of cells when grown overnight in culture tubes (see Fig. S1 in the supplemental material). A few strains did not exhibit enhanced biofilm formation in the presence of heparin (Fig. 1A, strains MW2 and UAMS1), and these strains aggregated poorly (Fig. S1).

10.1128/mSphere.00135-17.1 FIG S1  Heparin leads to aggregation of *S. aureus* in culture tubes. Various *S. aureus* strains were grown in the presence or absence of sodium heparin or ammonium heparin in culture tubes with shaking, as indicated. For each set of tubes, the tube treatments were as follows: no heparin (leftmost tube), sodium heparin (middle tube), and ammonium heparin (rightmost tube). The pictures are representative of the results from two independent experiments. Download FIG S1, TIF file, 2.4 MB. Copyright © 2017 Mishra and Horswill. 2017 Mishra and Horswill This content is distributed under the terms of the Creative Commons Attribution 4.0 International license.

Analysis of the effects of heparin on biofilm formation was further extended to coagulase-negative staphylococci (CoNS), including *Staphylococcus epidermidis*, *S. lugdunensis*, *S. hominis*, *S. haemolyticus*, *S. saprophyticus*, and *S. warneri*. There is increasing incidences of device-related infections of *S. epidermidis* and other related CoNS species (19), and thus, we extended our analysis of heparin impact to these CoNS. *S. epidermidis*, *S. haemolyticus*, and *S. hominis* showed a 90% to 400% increase in biofilm biomass with heparin supplementation, whereas *S. lugdunensis*, *S. warneri*, and *S. saprophyticus* remained unaffected under the conditions used (Fig. 1B). Taken together, these results suggest that heparin-mediated increase in biofilm biomass is a widespread phenomenon among various strains of *S. aureus* and CoNS.

To further investigate the heparin enhancement mechanism, a community-associated MRSA (CA-MRSA) isolate of the USA300 lineage (strain LAC) was used in this work. This strain was chosen because of its clinical significance and the availability of existing tools and a transposon mutant library (20). Reports of isolation of USA300 in biofilm infections such as infective endocarditis, osteomyelitis, and prosthetic joint infections have increased (21–23), suggesting these strains are relevant for study of biofilm mechanisms. As noted above, LAC biofilm capacity is enhanced with sodium or ammonium heparin (Fig. 1A), and further testing showed a dose-dependent increase in biofilm biomass with sodium heparin that plateaued at about 100 U/ml (Fig. 1C).

### Heparin does not impact *S. aureus* growth or pH.

A series of initial experiments were carried out to investigate the mechanism of heparin enhancement of biofilms. One possible mechanism is through heparin improvement of *S. aureus* growth, since bacteria have been observed to degrade and grow on heparin (24). However, heparin supplementation did not affect growth of the wild-type LAC strain (Fig. S2), suggesting that heparin impacts some phase of biofilm development. For *in vitro* studies, glucose is a known additive that positively impacts *S. aureus* biofilm formation (25), and this occurs through a decrease in the pH of the medium due to acetate excretion (26), repressing the *agr* quorum-sensing regulon (26). However, heparin supplementation in tryptic soy broth (TSB) did not lower the pH, even after biofilms were allowed to form for 18 h (Table 1). The only major changes in pH observed in the biofilm medium with various *S. aureus* strains were due to glucose supplementation.

10.1128/mSphere.00135-17.2 FIG S2  Heparin does not impact *S. aureus* growth. Growth of strain LAC in microtiter plates in the presence or absence of heparin, as indicated. The OD_600_ was determined every 15 min for 18 h. The results presented are representative of the results from three independent experiments. Download FIG S2, TIF file, 0.2 MB. Copyright © 2017 Mishra and Horswill. 2017 Mishra and Horswill This content is distributed under the terms of the Creative Commons Attribution 4.0 International license.

**TABLE 1:** Effect of heparin on pH

Strain	pH of medium^a^					
	TSB	TSB+AH	TSB+SH	TSB+G	TSB+G+AH	TSB+G+SH
None (control)	7.34	7.33	7.34	7.34	7.34	7.33
Wild-type LAC	6.86	6.85	6.94	4.89	4.91	4.89
Newman	6.59	6.67	6.63	4.88	4.76	4.93
RN6390	6.67	6.61	6.68	4.65	4.88	4.7
MN8	6.65	6.84	6.79	5.06	5.06	5.03
FRI 1161	6.69	6.79	6.72	5.02	5.01	5.02
UAMS-1	7.06	7.04	6.96	4.87	4.85	4.91
MW2	6.76	6.79	6.79	4.76	4.66	4.75
SH1000	6.9	6.82	6.75	4.98	4.96	4.96
JE2	6.72	6.79	6.78	4.97	5.02	4.96

a Tryptic soy broth (TSB) alone or supplemented with 100 U/ml of ammonium heparin (AH), 100 U/ml of sodium heparin (SH), and/or 0.2% glucose (G).

### Heparin increases biofilm cell retention but does not initiate attachment.

**FIG 2:**
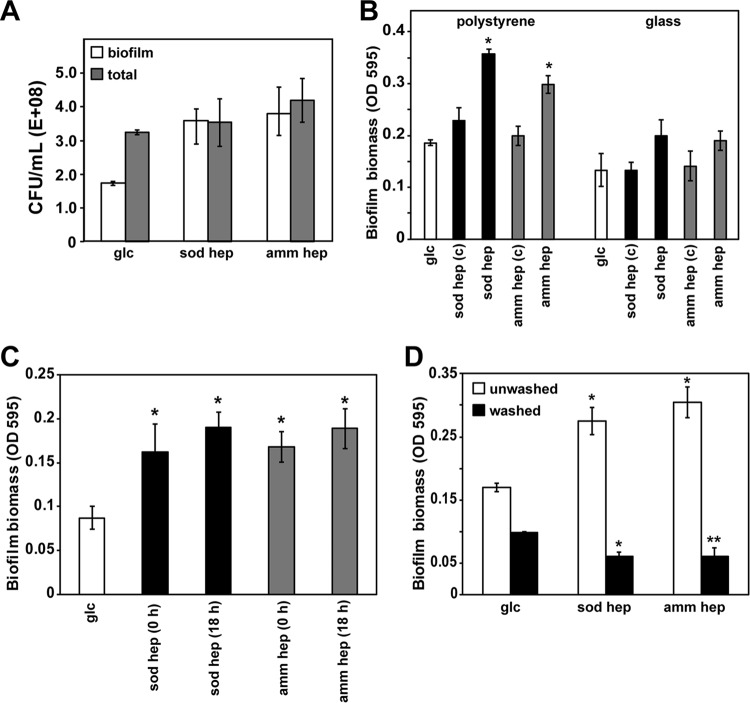
Heparin enhances cell retention within a biofilm but does not promote attachment. (A) The fraction of planktonic cells compared to biofilm cells was determined by suspending either the entire contents of a microtiter well (total) or just the biofilm after removal of spent medium (biofilm). When heparin was present, the number of planktonic cells was negligible compared to the number of cells in the biofilm. (B) The wells of a polystyrene or glass-bottom microtiter plate were incubated with 100 U/ml of heparin in PBS for 24 h [indicated by “(c)” (for coated)], washed to remove free heparin, and inoculated with strain LAC. After 18 h of incubation, biofilm biomass was assessed. As a negative control, wells were preincubated with PBS, and as a positive control, heparin was added at the time of inoculation with the LAC strain. (C) To assess the kinetics of heparin enhancement of biofilm formation, heparin was added at the time of inoculation (0 h) or after 18 h of growth in the absence of heparin (18 h). Biofilm biomass was determined after one additional hour of incubation. (D) Effect of washing on heparin-dependent biofilm formation. Strain LAC was grown for 18 h in the absence of heparin, at which time the cells were washed to remove secreted proteins and then transferred to the wells of a microtiter plate. Heparin was added, and biofilm biomass was assessed after 1 h. For comparison, the assay was also performed using the planktonic cells directly (unwashed). A glucose-only control (glc) received no heparin. Values in each panel are the averages ± standard deviations from three independent experiments. Statistical significance (*, *P* < 0.05; **, *P* < 0.005) was determined by a Student *t* test and is based on comparison to the value for the untreated control for each condition.

Biofilms consist of aggregates of bacteria attached to a surface and held together in a complex extracellular matrix (5, 27). We hypothesized that heparin may alter the properties of the matrix, leading to enhanced retention of cells that results in a thicker, more dense biofilm structure. To test this hypothesis, the number of CFU within the biofilm was determined and compared to the number of planktonic cells surrounding the biofilm, with and without heparin supplementation. Importantly, the number of CFU increased up to 50% in the presence of sodium heparin, while the total number of cells (both planktonic and biofilm) remained unaffected (Fig. 2A). This increase in biofilm biomass and CFU was independent of glucose supplementation (data not shown). These data suggest that heparin retains *S. aureus* cells within the biofilm matrix at the expense of neighboring planktonic cells.

Biofilm formation begins with bacterial attachment to a surface, and the positive impact of heparin on *S. aureus* biofilms could be due in part to improved cell attachment. This question was tested by coating microtiter wells with sodium heparin (100 U/ml), washing with phosphate-buffered saline (PBS) to remove free heparin, and then inoculating with strain LAC to initiate biofilm development. Control wells were set up in parallel with an identical concentration of heparin added to the medium at the time of inoculation. As shown in Fig. 2B, the heparin-dependent increase in biofilm biomass was observed only in the control wells, suggesting that heparin does not facilitate direct bacterial attachment to the microtiter plates. Similar results were obtained using glass plates (Fig. 2B), indicating that altering the surface chemistry does not change the observation. Taken together, heparin improves *S. aureus* cell retention within a biofilm but does not enhance surface attachment.

To further investigate these observations, biofilms were allowed to establish before heparin supplementation. Remarkably, heparin still increased biofilm biomass within 1 h of addition (Fig. 2C). This observation held true even if chloramphenicol was added to inhibit protein synthesis just prior to heparin addition (data not shown), suggesting that heparin does not induce production of biofilm-related proteins. Further support for this interpretation came from experiments in which *S. aureus* was grown for 18 h in culture tubes and transferred to the wells of a microtiter plate. The addition of heparin to these cell suspensions promoted biofilm formation within 1 h (Fig. 2D). If the cells were washed before transfer to the microtiter plate, heparin did not enhance biofilm formation (Fig. 2D). Washing not only affects the pH of the medium but also removes proteins from the extracellular environment. Collectively, these findings are consistent with a model in which heparin facilitates the rapid capture of planktonic cells from the surrounding liquid phase and incorporates them into the biofilm. The negative impact of washing suggests extracellular proteins might be involved, and this question is further addressed below.

### Heparin localizes within the biofilm matrix.

**FIG 3:**
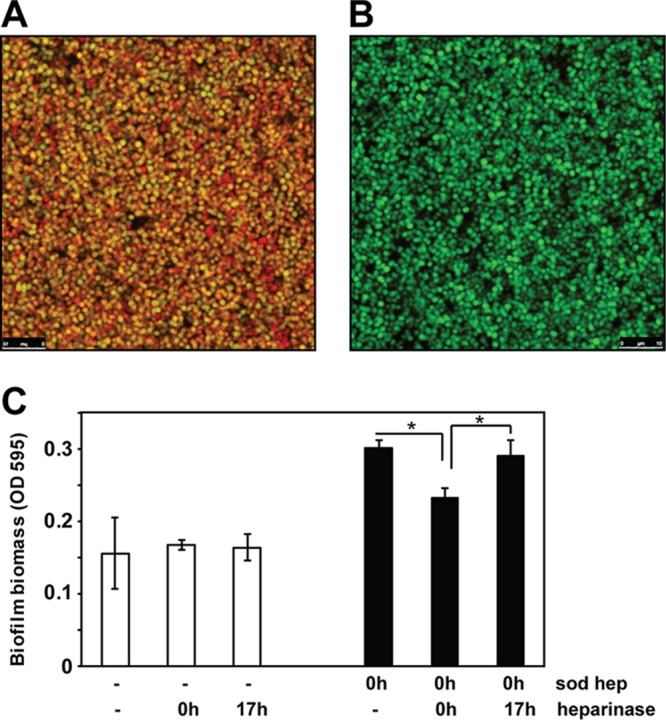
Heparin localizes in the *S. aureus* biofilm matrix. Confocal laser scanning microscopic images of LAC sGFP biofilm with (A) or without (B) rhodamine-heparin. (C) Effect of heparinase on biofilm biomass. Strain LAC was allowed to form biofilms for 18 h in the absence (−) (control) or presence of heparin. Where indicated, heparinase was added at the time of inoculation (0 h) or after allowing biofilms to form for 17 h. Values are the averages ± standard deviations from three independent experiments. Statistical significance (*, *P* < 0.05) was determined by a Student *t* test.

The model described above predicts that heparin is incorporated into the biofilm extracellular matrix. To test this idea, a green fluorescent protein (GFP)-expressing LAC strain was allowed to grow to stationary phase (18 h), at which time rhodamine-labeled heparin (rhodamine-heparin) was added. After an additional hour of incubation, biofilms were examined by confocal microscopy. As predicted, rhodamine-heparin was found to accumulate around the green fluorescent LAC strain (Fig. 3A). Biofilms that lacked exogenously added rhodamine-heparin showed only green fluorescence (Fig. 3B). The importance of heparin as a potential matrix component was further tested using heparinase (heparin lyase), which catalyzes breakdown of heparin into constituent disaccharides. Initially, heparinase was added together with heparin at the time of inoculation. In this case, heparinase limited the enhancement of biofilm formation by heparin (Fig. 3C), presumably due to breaking down before a positive impact on the biofilm could be observed. If heparin was added and the biofilm was allowed to establish, the addition of heparinase had no effect (Fig. 3C). In a mature biofilm, it is possible that heparin is inaccessible to the enzyme, or potentially an established biofilms is not affected by the removal of heparin from the matrix. With evidence that heparin is incorporated into the matrix, these observations raise questions of whether it substitutes for, or works together with, other components of the matrix.

### Heparin substitutes for eDNA in the biofilm matrix.

**FIG 4:**
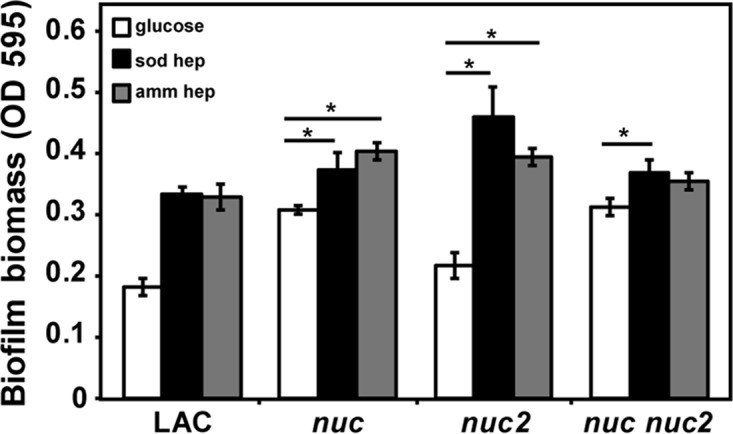
Effect of eDNA on heparin-mediated biofilm formation. Effect of heparin on biofilm formation in mutants defective in one or both nucleases (*nuc* and *nuc2*) was compared with the wild-type LAC strain. Three independent biological experiments were performed, each with four technical replicates, and the error bars represent the standard deviation of the entire data set. Statistical significance (*, *P* < 0.05) was determined by a Student *t* test.

*S. aureus* biofilms have an extracellular matrix that consists of polysaccharides, proteins, and eDNA (5, 27–30). Evidence in favor of eDNA as an important matrix component came in part from studies on the role of secreted nuclease in biofilm development. A mutant defective in the *nuc* gene, which encodes the major secreted nuclease in strain LAC, forms larger biofilms and accumulates more eDNA in the matrix than the wild type does (31, 32). Heparin is a large polyanionic polymer like eDNA and potentially could mimic the function of eDNA in the biofilm matrix. The observed heparin interaction with surface-localized proteins (Fig. 2D and 3), and positive impact on bacterial aggregation (Fig. S1), are consistent with this line of reasoning. Therefore, we hypothesized that heparin-dependent biofilm would be affected by modulating the level of eDNA in the matrix. To test this hypothesis, the effect of heparin on biofilm formation was assayed in nuclease mutants, which accumulate more eDNA than wild-type strains do (31). A LAC *nuc* mutant exhibited more-robust biofilms than the wild type did, consistent with previous reports (31), but the addition of heparin did still lead to a further increase in biofilm biomass (Fig. 4). Conversely, a LAC *nuc2* mutant, which lacks the additional surface-localized nuclease (33), formed a biofilm like the wild-type-like biofilm that was significantly enhanced by exogenous heparin. This observation is consistent with previous reports that mutations in *nuc2* are not linked to biofilm formation (33). When both *nuc* and *nuc2* were removed, maximizing eDNA levels in the matrix, heparin biofilm enhancement was reduced (Fig. 4) and no longer reached significance with ammonium heparin. These findings support a model in which heparin and eDNA can substitute for one another in the biofilm matrix, both leading to a positive impact on biofilm biomass.

### Proteins are required for heparin-dependent biofilm formation.

**FIG 5:**
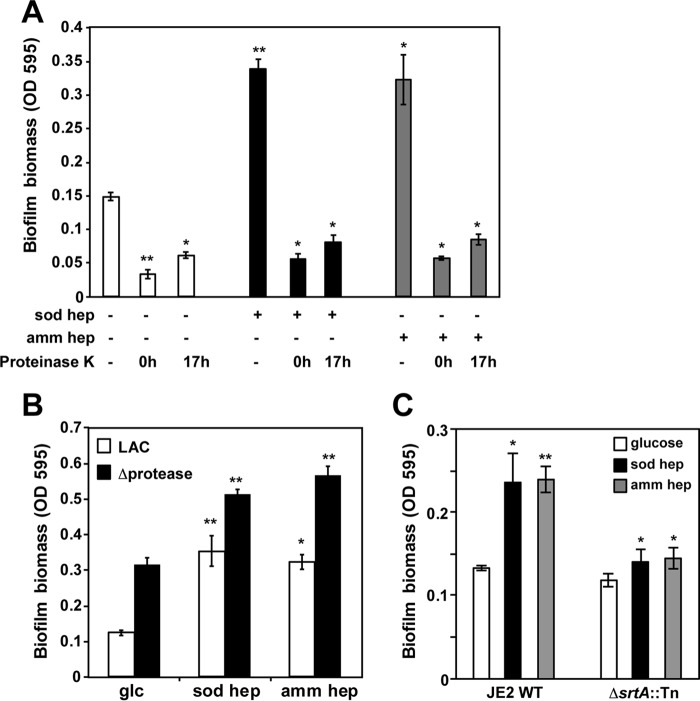
Proteins mediate heparin-dependent biofilm formation. (A) Proteinase K treatment abolishes biofilms whether added at the time of inoculation (0 h) or after allowing biofilms to form for 17 h. (B) Strain AH1919, a LAC derivative lacking 10 secreted proteases (Δprotease), forms a more robust biofilm than the wild-type parent LAC did. The biofilms for both strains were also compared in the presence of sodium heparin and ammonium heparin as indicated. (C) Wild-type (WT) strain JE2 biofilms were compared to *srtA*::Tn mutant in the presence or absence of sodium and ammonium heparin as indicated. For each panel, the values are the averages ± standard deviations from three independent experiments, each with four technical replicates. Statistical significance (*, *P* < 0.05; **, *P* < 0.005) was determined by a Student *t* test and is based on comparison to the value for the untreated control for each condition.

On the basis of the results of the washing experiments (Fig. 2D), we predicted that surface and/or extracellular proteins are involved in heparin biofilm enhancement. To address this question, the sensitivity of heparin biofilms to proteinase K treatment was tested. Notably, proteinase K treatment completely disintegrated the biofilm containing heparin, regardless of whether heparin was added at the time of inoculation or after biofilms had been allowed to form for 18 h (Fig. 5A). Proteinase K also disintegrated the biofilm in the control wells that lacked heparin, suggesting that the biofilm of the LAC strain is mostly held together by proteins, as shown previously (34, 35). This finding was further investigated using a LAC mutant lacking all secreted proteases (AH1919 strain, Δproteases) (36, 37), which formed a more robust biofilm than the wild-type strain did (Fig. 5B), presumably by eliminating self-cleavage of surface/extracellular proteins. The biofilm of the protease deficient strain was further increased by heparin addition. This observation suggests that heparin does not stimulate biofilm biomass by inhibiting secreted protease activity. To gain further information on the proteins involved in binding heparin, wild-type LAC (JE2 version) was compared to a knock out in sortase A (Fig. 5C). The *srtA*::Tn mutant still formed a biofilm with glucose, but the enhancement with heparin (either sodium or ammonium forms) was reduced compared to strain JE2, which suggests that sortase-anchored proteins may have some contribution to the heparin phenotype. Taken together, the heparin biofilm enhancement depends on *S. aureus* surface/extracellular proteins, and the phenotype is independent of known secreted proteases.

### Identification of heparin-binding proteins by mass spectrometry.

In order to identify the matrix proteins that interact with heparin, the wild-type LAC strain was grown in biofilm media containing 100 U/ml of sodium heparin for 18 h. Heparin-binding proteins were identified separately in the culture supernatant and cell wall fractions. Briefly, the culture was centrifuged to pellet the cells, and additional heparin was added to the supernatant fraction to capture secreted heparin-binding proteins. Protoplasts were prepared, and heparin was added to the supernatant to capture released cell wall proteins. Avidin-agarose beads were used to precipitate heparin from the supernatant and cell wall fractions, and associated proteins were eluted, separated by SDS-PAGE, and visualized by silver staining. Isolated bands were excised from the gel, digested with trypsin *in situ*, and analyzed by mass spectrometry. Using 15% sequence coverage as a cutoff, peptides from more than 40 secreted proteins and 100 cell wall proteins were identified (Table 2). Peptides corresponding to nine of these proteins appeared in both fractions. The majority of the proteins identified in the cell wall fraction were cytoplasmic; however, some of the cytoplasmic proteins have been reported to localize on the cell wall in the biofilms (38–40).

**TABLE 2:** Heparin-binding proteins identified by mass spectrometry

Fraction and ORF	Protein^a^	Peptide count^b^	Spectrum count^c^	% coverage^d^	Heparin-binding motif^e^
Secreted fraction					
SAUSA300_0955	Autolysin	34	137	40	None
SAUSA300_0113	Immunoglobulin G-binding protein A	22	31	45	None
SAUSA300_0032	Penicillin-binding protein 2′	17	53	34	MKKIKIAKKFHL
SAUSA300_1058	Alpha-hemolysin	18	39	55	NHNKKL
SAUSA300_0760	Enolase	6	17	27	None
SAUSA300_1150	Elongation factor Ts	12	5	16	GRLRKY
SAUSA300_0756	Glyceraldehyde-3-phosphate dehydrogenase	2	12	25	None
SAUSA300_0533	Elongation factor Tu	6	22	51	None
SAUSA300_2436	Surface protein G (putative cell wall surface anchor family protein)	17	32	15	LKRFHSRKN
SAUSA300_0055	Zinc-dependent alcohol dehydrogenase	2	23	51	None
SAUSA300_2579	*N*-acetyl-muramoyl-l-alanine amidase	8	17	17	None
SAUSA300_1790	Foldase protein PrsA precursor	8	14	23	VKSKKS
SAUSA300_0602	Uncharacterized protein	12	22	60	None
SAUSA300_0307	Lipoprotein family 5′-nucleotidase	9	51	35	IKKNKGAKKSHI
SAUSA300_0993	Pyruvate dehydrogenase E1 component alpha subunit	3	11	29	VRFRKF
SAUSA300_1975	Aerolysin/leukocidin family protein	12	30	41	None
SAUSA300_1974	Leukocidin/hemolysin	7	15	28	VHYKRS
SAUSA300_0536	Molecular chaperone Hsp31 and glyoxalase 3	2	13	31	None
SAUSA300_2079	Fructose bisphosphate aldolase	3	4	16	None
SAUSA300_1362	DNA-binding protein HU	3	12	56	None
SAUSA300_0099	1-Phosphatidylinositol phosphodiesterase	7	7	21	None
SAUSA300_0994	Pyruvate dehydrogenase E1 component beta subunit	4	11	43	None
SAUSA300_1382	LukS	4	7	17	None
SAUSA300_1381	LukF	7	9	30	AKKSKI
SAUSA300_0618	ABC transporter substrate-binding protein	10	5	16	None
SAUSA300_2573	IsaB	6	14	30	None
SAUSA300_2199	50S ribosomal protein L22	2	8	57	LKRFRPPRKVRL
SAUSA300_2189	50S ribosomal protein L6	2	7	29	None
SAUSA300_2506	Probable transglycosylase IsaA	5	12	35	None
SAUSA300_1603	50S ribosomal protein L21	4	3	39	None
SAUSA300_1920	Chemotaxis inhibitory protein	4	7	27	THHHSAKA
SAUSA300_2177	50S ribosomal protein L17	2	5	30	None
SAUSA300_1052	Fibrinogen-binding protein	3	3	21	FKRTRTTHRKAQRA
SAUSA300_0964	Chitinase	2	2	18	None
SAUSA300_2196	50S ribosomal protein L29	3	2	32	None
SAUSA300_0801	Enterotoxin, Seq	3	3	18	NKTKKGIKLRKY
SAUSA300_0883	Putative surface protein	4	5	42	None
SAUSA300_2195	30S ribosomal protein S17	2	2	24	YKTHKLGKRVKYSKKYKT
SAUSA300_0994	Putative pyruvate dehydrogenase E1 beta subunit	3	7	65	None
SAUSA300_2164	Surface protein	2	2	24	None
Cell wall fraction					
SAUSA300_0113	Immunoglobulin G-binding protein A	21	31	45	None
SAUSA300_2192	50S ribosomal protein L5	5	5	39	None
SAUSA300_0760	Enolase	16	17	27	None
SAUSA300_1150	Elongation factor Ts	13	5	16	GRLRKY
SAUSA300_0756	Glyceraldehyde-3-phosphate dehydrogenase	15	12	25	None
SAUSA300_0533	Elongation factor Tu	13	22	51	None
SAUSA300_0055	Zinc-dependent alcohol dehydrogenase	15	23	51	None
SAUSA300_0220	Formate acetyltransferase	13	16	21	LRSHKTYRKTHN
SAUSA300_2540	Fructose-bisphosphate aldolase class I	15	17	43	None
SAUSA300_1644	Pyruvate kinase	12	16	28	LKNKKGMRKTKI
SAUSA300_0757	Phosphoglycerate kinase	11	6	19	None
SAUSA300_1540	Chaperone protein DnaK	13	9	15	None
SAUSA300_0235	l-Lactate dehydrogenase	20	7	36	None
SAUSA300_2198	30S ribosomal protein S3	6	8	36	LKIRKF
SAUSA300_0532	Elongation factor G	15	16	32	IKKNKG|SRRGRVGRIHKI
SAUSA300_1201	Glutamine synthetase	10	9	16	ARKHNLHA
SAUSA300_1640	Isocitrate dehydrogenase	5	7	18	None
SAUSA300_2362	2,3-Bisphosphoglycerate-dependent phosphoglycerate mutase	8	20	52	None
SAUSA300_0536	Molecular chaperone Hsp31	10	13	31	None
SAUSA300_1138	Succinyl-CoA synthetase, beta subunit	11	7	18	None
SAUSA300_1149	30S ribosomal protein S2	5	10	18	PKMKKY
SAUSA300_0186	Phosphate acetyltransferase	10	11	41	None
SAUSA300_1365	30S ribosomal protein S1	13	7	15	None
SAUSA300_1641	Citrate synthase II, GltA	10	7	15	None
SAUSA300_0523	50S ribosomal protein L1	4	13	28	None
SAUSA300_2078	UDP-*N*-acetylglucosamine 1-carboxyvinyltransferase	5	5	17	GRFKKC
SAUSA300_1331	Alanine dehydrogenase	5	5	15	None
SAUSA300_0973	Phosphoribosylformylglycinamide cycloligase	6	7	22	None
SAUSA300_2362	2,3-Bisphosphoglycerate-independent phosphoglycerate mutase	10	13	24	None
SAUSA300_0389	GMP synthase	7	7	20	IKSHHN
SAUSA300_2178	DNA-directed RNA polymerase	9	11	36	None
SAUSA300_0129	2-Butanediol dehydrogenase	8	19	50	None
SAUSA300_1239	Transketolase	9	9	21	None
SAUSA300_0539	Branched-chain amino acid transferase	8	11	23	None
SAUSA300_0758	Triose phosphate isomerase	11	20	56	None
SAUSA300_1666	30S ribosomal protein S4	4	6	25	ARTRRQ
SAUSA300_0871	Fumarylacetoacetate hydrolase	7	7	35	None
SAUSA300_0886	3-Oxoacyl-synthase 2	8	10	33	None
SAUSA300_0067	Universal stress protein	4	10	34	None
SAUSA300_1491	Xaa-Pro dipeptidase	5	7	16	None
SAUSA300_1804	Glucosamine-6-phosphate isomerase	7	8	16	None
SAUSA300_1622	Trigger factor	5	6	15	None
SAUSA300_0966	N5-carboxyaminoimidazole ribonucleotide	6	5	39	None
SAUSA300_2092	DNA protection during starvation protein	6	14	58	None
SAUSA300_1976	Succinyl diaminopimelate desuccinylase	10	10	24	None
SAUSA300_0976	Phosphoribosylamine-glycine ligase	8	8	25	None
SAUSA300_2462	NAD(P)H-flavin oxidoreductase	4	5	19	None
SAUSA300_0491	Cysteine synthase	9	11	47	None
SAUSA300_0965	FolD	6	4	19	None
SAUSA300_1080	FtsZ	10	12	32	None
SAUSA300_1367	Cytidylate kinase	3	3	16	None
SAUSA300_1696	d-Alanine aminotransferase	6	5	22	None
SAUSA300_0860	Ornithine aminotransferase	6	6	21	None
SAUSA300_1880	Glutamyl-tRNA amidotransferase	6	6	15	None
SAUSA300_0234	Putative flavohemoprotein	11	7	25	None
SAUSA300_0135	Superoxide dismutase	6	9	43	None
SAUSA300_2067	Serine hydroxymethyltransferase	7	8	21	None
SAUSA300_0948	Naphthoate synthase	5	4	16	None
SAUSA300_0141	Phosphopentomutase	6	5	18	None
SAUSA300_1657	Acetate kinase	8	10	28	None
SAUSA300_2187	30S ribosomal protein S5	5	12	42	GRRFRF
SAUSA300_1725	Transaldolase	4	6	24	None
SAUSA300_2091	Purine nucleoside phosphorylase Deo-D type	4	5	31	None
SAUSA300_0009	Serine-tRNA ligase	5	8	18	None
SAUSA300_1874	Ferritin	7	11	40	None
SAUSA300_2202	50S ribosomal protein L23	4	5	46	None
SAUSA300_2517	Amidohydrolase family protein	4	4	17	None
SAUSA300_2463	d-Lactate dehydrogenase	7	7	20	None
SAUSA300_1615	Delta-aminolevulinic acid dehydratase	3	3	15	None
SAUSA300_2190	30S ribosomal protein S8	2	3	24	None
SAUSA300_1139	Succinyl-CoA synthetase, alpha subunit	5	5	26	GRKTRL
SAUSA300_0531	30S ribosomal protein S7	2	3	19	None
SAUSA300_0672	MarR family transcriptional regulator	3	3	30	None
SAUSA300_0688	Oxidoreductase/aldo-keto reductase family	6	6	23	None
SAUSA300_1109	Methionyl-tRNA formyl transferase	5	5	21	None
SAUSA300_0114	SarS	4	4	17	None
SAUSA300_1653	Metal-dependent hydrolase	3	4	21	None
SAUSA300_0916	Conserved hypothetical protein	6	6	37	None
SAUSA300_0605	SarA	4	4	31	None
SAUSA300_1442	SrrA	4	4	26	THVKRL
SAUSA300_0380	AhpC	5	8	40	AHKIKA
SAUSA300_2097	Uncharacterized protein	3	5	16	None
SAUSA300_1494	LipM	3	3	17	None
SAUSA300_1719	Arsenate reductase	2	2	20	None
SAUSA300_1900	Manganese-dependent inorganic pyrophosphatase	4	4	17	None
SAUSA300_1541	GrpE	3	3	17	None
SAUSA300_1191	Complement inhibitor	2	2	27	None
SAUSA300_0540	HAD family hydrolase	5	6	21	None
SAUSA300_0173	Uncharacterized protein	2	2	17	None
SAUSA300_0969	PurS	4	4	59	None
SAUSA300_1131	30S ribosomal protein S16	3	3	49	None
SAUSA300_1304	Glyoxylase family protein	3	3	18	None
SAUSA300_2529	PhnB	2	2	19	None
SAUSA300_2245	SarR	2	2	18	None
SAUSA300_2315	Lipoprotein	2	2	19	None
SAUSA300_1659	Probable thiol peroxidase	3	3	28	None
SAUSA300_0015	50S ribosomal protein L9	2	2	17	None
SAUSA300_2132	Uncharacterized protein	2	2	37	None
SAUSA300_1358	Nucleoside diphosphate kinase	2	2	15	None

a CoA, coenzyme A; HAD, haloacid dehalogenase.

b The peptide count is the number of exclusive unique peptides that matched the identified protein across all MS samples.

c The spectrum count is the number of counts of spectra that match different peptides (even if the peptides overlap), two different charge states of the same peptide, or both a peptide and a modified form of the peptide.

d % coverage is the percentage of all the amino acids in the protein sequence that were detected in the sample.

e Heparin-binding motif refers to the three linear heparin-binding motifs, namely, Cardin (XBBXBX), Weintraub (XBBBXXBX), and Sobel (XBBBXXBBBXXBBX).

The most abundant protein in the secreted fraction was bifunctional autolysin (Atl), which is the major peptidoglycan hydrolase in *S. aureus* (41). Atl was followed by protein A in the secreted fraction, and protein A was the most abundant protein in the cell wall fraction. Protein A is a cell wall-anchored (CWA) protein that is shed at high levels (42, 43), and it contains four or five homologous modules that can bind to multiple ligands (44). Apart from protein A, four other cell wall-anchored proteins were detected in the secreted fraction: penicillin-binding protein 2′ (PBP2′), SasG (SAUSA300_2436), a putative surface protein (SAUSA300_0883), and another surface protein (SAUSA300_2164). SasG is a known CWA surface protein that promotes biofilm formation by *S. aureus* (45). Interestingly, SasG (SAUSA300_2436) is truncated after 444 amino acids in the LAC strain, while SasG in other strains is intact at ~1,630 amino acids, depending on the strain. PBP2′ is present only in the MRSA strains and has been observed to affect biofilm formation in MRSA strain BH1CC (46). SAUSA300_2164 and SAUSA300_0883 have not been studied, but their heparin binding ability suggests that they probably increase cell-cell interaction by binding to heparin.

Detection of a few CWA proteins in the secreted fraction could be due to cell lysis. However, CWA proteins were not detected in the cell wall fraction. It is plausible that the large size (>100 kDa) might prevent the proteins from being captured in the heparin-binding assay. Moreover, CWA proteins have been reported to be low during planktonic growth compared to the immature biofilms (47). Also, high protease activity during the stationary phase of growth would likely result in their cleavage and localization in the secreted fraction than in the cell wall fraction.

Five cytoplasmic proteins were among the top 10 heparin-binding proteins in both the secreted and cell wall fractions: enolase, elongation factor Ts, glyceraldehyde-3-phosphate dehydrogenase (GAPDH), elongation factor Tu, and a Zn-dependent alcohol dehydrogenase (Table 2). Although cytoplasmic proteins in the cell wall fraction could be the result of contamination, recent studies suggest that this is not the case. Cytoplasmic proteins such as GAPDH and enolase have been reported to be a main constituent of the *S. aureus* biofilm matrix (38). These proteins reversibly associate with the cell surface at low pH and interact with eDNA to form a network of cells within the matrix (48). Additionally, nucleic acid-binding proteins such as IsaB (49), HU, elongation factors, and ribosomal proteins were detected in the secreted and cell wall fractions, consistent with the hypothesis that heparin can substitute for eDNA in the biofilm matrix.

There are some reports of toxins contributing to cell-cell interactions. The pore-forming cytotoxin alpha-hemolysin (Hla) has a role in *S. aureus* biofilm formation (50) and was identified in high levels of the secreted fraction (Table 2). Additional heparin-binding toxins included Panton-Valentine leukocidin (LukSF), enterotoxin Q, and LukG. The signal peptide of LukSF has been reported to act as a bridge between *S. aureus* and the heparin/heparan sulfate component of host extracellular matrix (ECM) *in vivo* (51). Although this signal peptide was not detected specifically in the heparin-binding assay, it is likely that the processed proteins possess a heparin-binding property as well.

The appearance of multiple proteins with diverse physiological roles in the heparin-bound fraction suggests that the interaction between heparin and protein is somewhat nonspecific. However, heparin-binding motifs have been identified in some proteins from higher organisms, and these motifs consist of a linear array of basic and hydrophobic amino acids. Accordingly, the heparin-binding proteins identified here were scanned for three well-characterized heparin-binding motifs (HBMs) that have been identified in eukaryotic proteins, namely, the Cardin, Weintraub, and Sobel motifs (52, 53). Approximately 50% of the heparin-bound proteins from *S. aureus* possessed one or more of these HBMs (Table 2). The absence of obvious HBMs in many staphylococcal proteins with heparin-binding ability suggests that they may contain motifs different from previously defined eukaryotic HBMs.

### Analysis of mutants lacking individual heparin-binding proteins for their ability to form heparin-dependent biofilms.

Seventeen of the 40 heparin-binding proteins identified from the secreted fraction were tested for their significance in follow-up biofilm assays using insertion mutants from the Nebraska Transposon Mutant Library (15). These 17 mutants were chosen because they are known or predicted to be either secreted or surface exposed, and transposon mutants were available in the library. All 17 of the mutants still responded to heparin by forming an enhanced biofilm, but in several cases, the magnitude of the response was relatively modest (Table 3). For example, whereas exogenous heparin increased biofilm biomass of the wild-type LAC strain by 80 to 100%, more modest increases of 20 to 40% were observed from mutants lacking IsaB, *N*-acetylmuramoyl-l-alanine amidase, Panton-Valentine leukocidin, LukS-PV, and a leukocidin/hemolysin toxin family protein (SAUSA300_1974) (Table 3). The fact that none of the mutants assayed completely lost the ability to respond to heparin suggests that there is redundancy in the binding ability. Additionally, essential cytoplasmic proteins such as GAPDH and enolase, whose mutants were not available in the transposon library, might play an important role in heparin-containing biofilms.

**TABLE 3:** Effect of heparin on biofilm biomass formation by transposon mutants of *S. aureus* strain USA300_FPR3757

FPR3757 no.	Protein (no. of amino acids)	Biofilm biomass		
		No heparin (% change in biomass compared to LAC)^a^	Sodium heparin [OD_595_ ± SD (% change in biomass compared to no heparin)^a^]	Ammonium heparin [OD_595_ ± SD (% change in biomass compared to no heparin)^a^]
SAUSA300_0955	Autolysin (1,256)	0.0241 ± 0.013 (−65)	0.046 ± 0.004 (92)	0.038 ± 0.005 (57)
SAUSA300_0113	Immunoglobulin G-binding protein A (508)	0.132 ± 0.015 (13)	0.246 ± 0.041 (85)	0.269 ± 0.018 (103)
SAUSA300_0032	Penicillin-binding protein 2′ (668)	0.147 ± 0.02 (11.4)	0.332 ± 0.008 (126)	0.257 ± 0.023 (75)
SAUSA300_1058	Alpha-hemolysin precursor (319)	0.176 ± 0.029 (27)	0.282 ± 0.025 (60)	0.248 ± 0.019 (41)
SAUSA300_2436	Putative cell wall surface anchor family protein (444)	0.166 ± 0.006 (−2.6)	0.245 ± 0.023 (48)	0.253 ± 0.021 (52)
SAUSA300_2579	*N*-acetylmuramoyl-l-alanine amidase domain protein (619)	0.205 ± 0.01 (10.7)	0.274 ± 0.016 (34)	0.248 ± 0.012 (21)
SAUSA300_0602	Hypothetical protein (168)	0.171 ± 0.03 (37)	0.254 ± 0.008 (49)	0.25 ± 0.015 (47)
SAUSA300_0307	5′-Nucleotidase (296)	0.163 ± 0.003 (−13.4)	0.244 ± 0.03 (49.4)	0.292 ± 0.026 (79)
SAUSA300_1975	Aerolysin/leukocidin family protein (351)	0.123 ± 0.009 (−32)	0.022 ± 0.009 (79)	0.21 ± 0.01 (72)
SAUSA300_1974	Leukocidin/hemolysin toxin family protein (338)	0.182 ± 0.02 (−0.76)	0.237 ± 0.023 (30)	0.245 ± 0.023 (35)
SAUSA300_0099	1-Phosphatidylinositol phosphodiesterase, Plc (328)	0.153 ± 0.03 (−4.37)	0.309 ± 0.04 (102)	0.297 ± 0.021 (94)
SAUSA300_1382	Panton-Valentine leukocidin, LukS-PV (312)	0.18 ± 0.015 (22)	0.248 ± 0.023 (37)	0.234 ± 0.018 (29)
SAUSA300_0618	ABC transporter substrate-binding protein (309)	0.149 ± 0.009 (−3.04)	0.289 ± 0.023 (93)	0.234 ± 0.016 (56)
SAUSA300_2573	Immunodominant antigen B (175)	0.253 ± 0.016 (24)	0.308 ± 0.021 (21)	0.303 ± 0.014 (19)
SAUSA300_1920	Chemotaxis-inhibiting protein CHIPS, Chs (149)	0.161 ± 0.002 (−17)	0.286 ± 0.021 (78)	0.228 ± 0.017 (41)
SAUSA300_0801	Staphylococcal enterotoxin Q, *seq* (242)	0.118 ± 0.023 (−5)	0.215 ± 0.015 (82)	0.186 ± 0.009 (58)
SAUSA300_0883	Putative surface protein (144)	0.176 ± 0.018 (3.46)	0.311 ± 0.02 (77)	0.261 ± 0.01 (49)

a Percentages of biofilm biomass for each transposon mutant defective in respective protein in the presence of heparin relative to that lacking heparin represent the average data (±SD) from three biological replicates. Each biological replicate value was the average of three technical replicates.

## DISCUSSION

Staphylococci are the dominant pathogens associated with indwelling medical device infections (19). A characteristic feature of these infections is the formation of biofilms, which are recalcitrant to antibiotic treatment and host immune responses. It was previously reported that sodium heparin used in catheter lock solutions stimulates *S. aureus* biofilm formation, but the mechanism of this enhancement has remained elusive (9). Many surface and regulatory factors contributing to *S. aureus* biofilms have been identified in the last 2 decades (5, 27, 54), and some of the common biofilm determinants identified (e.g., *agr* system, SarA, and polysaccharide) do not play a significant role in the heparin-mediated increase (9). In this work, we sought to obtain a more complete understanding of the positive impact of heparin on staphylococcal biofilm development. Previous work in the lab showed that a nonsulfated glycosaminoglycan, hyaluronan, is incorporated into the *S. aureus* matrix and promotes biofilm formation (10). Although heparin differs from hyaluronan in being highly negatively charged, they are structurally similar glycosaminoglycans. Heparin resembles another matrix component, eDNA, in being a polyanion, and therefore, we hypothesized that heparin would also be incorporated into the biofilm matrix. Indeed, we observed heparin incorporation into the *S. aureus* biofilm matrix (Fig. 3A). These biofilms remained proteinase K labile, suggesting that matrix proteins play a significant role in the heparin-dependent enhancement phenotype.

We used a pulldown assay coupled with proteomics to identify multiple *S. aureus* heparin-binding proteins from both the secreted and cell wall fractions. Interestingly, most of these proteins have been either identified or proposed as DNA-binding proteins. Atl was the most abundant heparin-bound protein in the secreted fraction (Table 2), and it is a bifunctional autolysin that contributes to biofilm formation by release of cytoplasmic proteins and eDNA through cell lysis (41, 55, 56). Atl has been shown to bind to eDNA and host proteins such as fibrinogen, fibronectin, and vitronectin, which often coat the surfaces of biomedical devices (57, 58). Two other abundant DNA-binding proteins identified were IsaB and HU. IsaB nonspecifically binds to nucleic acids (49), and this protein was isolated from both the secreted and cell wall fractions in the heparin-binding assay. HU is an essential histone-like DNA-binding protein, which is capable of wrapping DNA to protect it from denaturation during extreme environmental conditions. Since heparin structurally resembles the eDNA component of the biofilm matrix in being a polyanion, we propose that heparin can substitute for eDNA during *S. aureus* biofilm formation. One of the best-characterized eDNA-binding proteins is beta toxin (59), which was not detected in the heparin-binding assay. However, the LAC strain and many other *S. aureus* strains are reported to contain a bacteriophage (φSa3) integrated into the structural gene, *hlb*, rendering it ineffective (60, 61). Support for our hypothesis that heparin substitutes for eDNA also came from the analysis of mutants defective in nucleases. These mutants contain higher levels of eDNA in the matrix and form more-robust biofilms than the wild type does (31–33). Importantly, biofilm capacity of these mutants was not enhanced further by the addition of heparin (Fig. 4), suggesting that there is likely enough eDNA present to saturate binding sites. Taken together, these observations demonstrate that heparin mimics eDNA and binds to proteins that normally associate with eDNA during biofilm development.

Our findings also support observations by Foulston and coworkers and Dengler and coworkers in explaining the contribution of cytoplasmic proteins, such as GADPH and enolase, to *S. aureus* biofilm formation (38, 48). According to their proposed model, these cytoplasmic proteins become positively charged at low pH (~5) due to acidification during growth on glucose, and the proteins interact with the negatively charged cell surface and eDNA to form bacterial aggregates. The eDNA is thought to tether bacterial cells together in the matrix, leading to enhanced biofilm formation (62). In the present work, our heparin-binding assay identified the same GAPDH and enolase proteins in both the secreted fraction and the cell wall fraction (Table 2). Additionally, heparin-dependent biofilm formation was inhibited when the pH of the medium was altered by washing stationary-phase cells (Fig. 2D), suggesting a similar pH-dependent biofilm phenotype. Our observations suggest that heparin can interact with the same negatively charged cytoplasmic proteins, capturing and tethering cells together in the matrix and thereby promoting *S. aureus* biofilm formation.

Although multiple heparin-binding proteins were identified in this work, the nature of their interaction with heparin is not clear. The multiplicity of heparin-binding proteins suggests a relatively nonspecific interaction, such as an electrostatic interaction between two oppositely charged biomolecules. Literature reports on heparin-protein interaction lack information on the structural requirements of a protein for such an interaction (63, 64). Three different amino acid sequence-based motifs that contain basic and hydropathic amino acid residues in a certain order have been proposed on the basis of a few well-characterized heparin-protein interactions (52, 53). It has been suggested that positively charged residues in a protein interact with negatively charged carboxylate and sulfate ions of heparin; however, these interactions did not seem to impart specificity. Consistent with this notion, only about half of the heparin-binding proteins identified in this study had heparin-binding motifs (HBMs). These motifs were absent in key cytoplasmic proteins identified, including GAPDH, enolase, and elongation factor Tu. Also, a well-characterized nucleic acid-binding protein, IsaB, which binds to heparin, lacked any linear HBMs, which suggests that these linear HBMs are only one component of the heparin-mediated increase in biofilm formation.

The widespread usage of heparin and increasing incidence of catheter-related infections suggest the need for alternative lock solutions. An ideal lock solution should prevent thrombosis and inhibit bacterial and fungal infections. Trisodium citrate (4%) with antithrombotic efficacy similar to heparin has emerged as an alternative. The antithrombotic property of citrate is exerted by Ca^2+^ chelation (65). Chelation of divalent metal ions such as Ca^2+^ and Mg^2+^ also helps to prevent bacterial colonization (66) and makes citrate a suitable alternative to heparin as a lock solution. Nevertheless, side effects due to citrate usage have limited its widespread implementation. Other alternatives to heparin include ethanol and taurolidine/citrate; however, heparin still remains the antithrombotic chemical of choice in hospital settings. In order to avoid device-related infections, vancomycin is currently added to heparin lock solution; however, the emergence of vancomycin-resistant *S. aureus* (VRSA) makes the usage of antimicrobial agents less reliable. Results of this work could guide future strategies to combat heparin-stimulated biofilm formation on indwelling devices.

## MATERIALS AND METHODS

### Bacterial strains and growth conditions.

Bacterial strains used in this work are listed in Table 4. *S. aureus* and coagulase-negative staphylococcal (CoNS) strains were routinely grown in tryptic soy broth (TSB) or on tryptic soy agar (TSA) plates. For microtiter plate-based biofilm assay, *S. aureus* and CoNS strains were grown in 66% TSB (20 g/liter of TSB) supplemented with 0.2% glucose at 37°C with shaking at 200 rpm. Sodium heparin (catalog no. H3393; Sigma-Aldrich, St. Louis, MO) (100 U/ml) or ammonium heparin (catalog no. H6279; Sigma-Aldrich, St. Louis, MO) (100 U/ml) was added to the cultures where mentioned. When appropriate, the following antibiotics were added to the culture at the concentrations indicated: spectinomycin (1,000 μg/ml) and erythromycin (10 μg/ml).

**TABLE 4:** Bacterial strains used in this study

Strain	Description	Reference(s) or source
AH1263	*S. aureus* USA300 CA-MRSA Erm^s^ (LAC)	71
JE2	*S. aureus* CA-MRSA USA300 Erm^s^; plasmid-cured LAC derivative	20
AH0204	*S. aureus* Newman (MSSA strain)	72
AH0206	*S. aureus* RN6390 (*agr*^*+*^ laboratory strain related to strain 8325-4 but with a defective *rsbU*)	73
AH0247	*S. aureus* MN8 (clinical isolate from menstrual toxic shock syndrome case)	74
AH0248	*S. aureus* FRI1169	
AH0386	*S. aureus* SH1000 (functional *rsbU* derivative of strain 8325-4)	75
AH0411	*S. aureus* UAMS-1 (MSSA USA200 osteomyelitis isolate)	76
AH0843	*S. aureus* MW2 (USA400 CA-MRSA; clinical isolate from a necrotizing pneumonia case)	77
AH1359	*S. aureus* AH1263 containing pCM12 (an *Escherichia coli-S. aureus* shuttle vector expressing superfolder GFP) Spec^r^	78, 79
AH1680	*S. aureus* USA300 CA-MRSA Erm^s^ (LAC) *nuc*::LtrB	31
AH3051	*S. aureus* AH1263 *nuc*::LtrB *nuc2*::*erm*	33
AH3057	*S. aureus* AH1263 *nuc2*::*erm*	33
AH1919	*S. aureus* LAC Δ*aur* Δ*sspA* Δ*scpA* Δ*spl*::*erm*	36
AH1738	*S. epidermidis* 1457	80
BB2191	*S. lugdunensis*	81
BB2201	*S. warneri*	P. Schlievert
BB2203	*S. hominis*	P. Schlievert
BB2205	*S. haemolyticus*	P. Schlievert
BB2153	*S. saprophyticus* 7108	ATCC

### Microtiter plate-based biofilm assay.

Biofilms were routinely grown in 96-well flat-bottom polystyrene microtiter Costar plates (catalog no. 3595; Corning Inc., Corning, NY). Where specified, glass microtiter plates were used instead (Costar [catalog no. 3631; Corning Inc.]). Briefly, *S. aureus* cultures grown overnight in TSB were diluted 1:200 in 66% TSB supplemented with 0.2% glucose. Sodium heparin or ammonium heparin (100 U/ml) was added to each experimental well wherever mentioned. Where stated, additional compounds were added to the biofilm growth condition at the following concentration: 25 U/ml of DNase I, 100 μg/ml of proteinase K, and 30 μg/ml of chloramphenicol. Microtiter plates for the biofilm assay were maintained at 37°C with shaking at 200 rpm for the indicated number of hours and assayed for biofilm formation using a crystal violet staining method (28). For measurement of biofilm, cell suspension was removed from the wells prior to washing twice with 0.2 ml of 0.02 M phosphate-buffered saline (PBS). Each biofilm in a well was stained with 0.1 ml of crystal violet (0.1% [wt/vol] in water) for 10 min at room temperature. The plates were inverted and photographed with a Canon EOS Rebel digital camera. Biofilm biomass was measured as the optical density at 595 nm (OD_595_) after solubilization of crystal violet stain with acidified ethanol (40 mM HCl in ethanol) using a Tecan Infinite 200 Pro microtiter plate reader (Tecan Trading AG, Switzerland).

### Attachment to the microtiter plate surface.

The effect of heparin on bacterial attachment was assayed in both polystyrene and glass microtiter plates. Briefly, wells were pretreated with 200 μl of 100 U/ml or 500 U/ml of heparin in 0.02 M PBS, pH 6.8, for 24 h at 37°C without shaking. Control wells contained only 0.02 M PBS. After 24 h, all the wells were washed twice with 0.02 M PBS, and biofilm assays were set up as described above. The attachment assay was also carried out on a glass surface using 96-well glass-bottom microtiter plates (catalog no. C3631; Corning Inc., Corning, NY).

### Measurement of growth kinetics and cell viability.

Overnight cultures were diluted 1:200 in TSB and grown until the OD_600_  reached 0.1 (log phase), then diluted 1:200 into 66% TSB supplemented with 0.2% glucose. Two hundred microliters of culture was dispensed into each well of the 96-well flat-bottom microtiter plate. Sodium heparin or ammonium heparin (100 U/ml) was added to each experimental well, and growth was monitored as OD_600 _using a Tecan Infinite 200 Pro microtiter plate reader (Tecan Trading AG, Switzerland). Final growth achieved in the biofilm medium was also measured as the total number of CFU present in the biofilm medium after 18 h. To do this, bacterial culture in each well was mixed properly by pipetting to dislodge the biofilm. Proteinase K (100 μg/ml) was added to each well for 15 min at room temperature to disintegrate the biofilm biomass. Proteinase K-treated bacteria were taken out in a microcentrifuge tube and vigorously vortexed to further remove aggregates prior to dilution into TSB. Appropriate dilutions were made in TSB and spread on TSA plates. The plates were incubated overnight at 37°C for colonies to appear, and the number of CFU was counted. In order to measure the number of CFU in biofilm, bacterial suspension containing planktonic cells was removed from each well. The biofilm biomass in each well was washed twice with sterile 0.02 M PBS and resuspended in 0.2 ml TSB and 100 μg/ml of proteinase K. CFU was determined as mentioned above.

### Confocal microscopy.

Confocal microscopy was performed with a Leica SP8 STED superresolution microscope (Leica Microsystems, Inc., Wetzlar, Germany). For imaging, an overnight culture of* S*. *aureus* AH1359, a derivative of wild-type USA300 expressing superfolder green fluorescent protein (sGFP) was grown in TSB supplemented with 200 μg/ml of spectinomycin (78, 79). This culture was diluted 200-fold in biofilm medium (66% TSB supplemented with 0.2% glucose), and 0.2 ml of culture was dispensed into each chamber of an eight-well chambered cover glass (Lab-Tek chambered 1.0 borosilicate cover glass system; Nunc, Rochester, NY). Bacteria were allowed to grow at 37°C for 18 h. After 18 h of growth, 0.5 mg/ml of rhodamine-labeled heparin (rhodamine-heparin) (catalog no. HP-204; Creative PEGWorks) was added to each test well, and incubation was continued for another hour at 37°C. Liquid media containing planktonic cells was removed, and biofilms were washed twice with 0.2 ml of 0.02 M PBS. Twenty microliters of PBS was added to each well to prevent cells from dehydrating, and biofilms were analyzed by confocal microscopy. An equal volume of 0.02 M PBS was added to each control well in place of rhodamine-heparin. The competition assay was also performed with the addition of rhodamine-heparin to the wells containing biofilm grown in the presence of 100 U/ml of sodium heparin.

### Sample preparation for mass spectrometry.

Overnight-grown USA300 wild-type culture in TSB was diluted 200 times in 100 ml of biofilm medium (66% TSB supplemented with 0.2% glucose) and grown at 37°C for 18 h with shaking at 200 rpm. Sodium heparin (200 U/ml) was added to cultures grown for 18 h and incubated for an hour. The cultures were pelleted, and supernatant was concentrated using a 10-kDa-cutoff Amicon Ultra-15 centrifugal filter unit (EMD Millipore, Billerica, MA). Concentrated supernatant was buffer exchanged with 0.02 M PBS, pH 6.8, using Amicon Ultra 0.5-ml filter units. Protein samples were also prepared from the pellet fraction. The pellets were washed twice with 0.02 M PBS, pH 6.8, and resuspended to an OD_600_ of 30 in a lysis buffer (50 mM Tris-HCl and 20 mM MgCl_2 _[pH 7.5] supplemented with 30% raffinose). Briefly, cell pellets were incubated in a lysis buffer containing 200 μg/ml of lysostaphin, 20 μg/ml DNase I, and protease inhibitor (mini Complete; Roche Molecular Biochemicals) for 30 min. Cell suspension was centrifuged at 6,000 × *g* for 30 min at 4°C. The supernatant fraction was further concentrated by Amicon Ultra 0.5-ml filter units.

### Peptide identification by mass spectrometry.

Portions (0.2 ml) of the samples were incubated with 0.1 ml of avidin-agarose beads (Thermo Scientific) for 30 min on a rocker at room temperature in the presence of 100 U/ml of sodium heparin. The beads were pelleted gently at 2,000 rpm for 2 min, and supernatant was withdrawn. The beads were then washed three times with 0.02 M PBS, pH 6.8, to further remove the unbound proteins. The bound fraction was eluted with 0.1 ml of 3 M NaCl. Eluted samples were pooled and buffer exchanged with 0.02 M PBS, pH 6.8, using a 10-kDa-cutoff Amicon Ultra 0.5-ml centrifugal filter unit before analyzing on SDS-polyacrylamide gels. The protein concentration in the sample was determined by the Bradford assay. Five micrograms of sample was mixed with 20 μl lithium dodecyl sulfate (LDS) buffer (pH 8.4), divided into four fractions, and loaded on NuPage 4 to 12% Bis-Tris precast gels (Invitrogen, Carlsbad, CA). Two peripheral lanes were loaded with Sharp prestained protein ladder standards (Invitrogen, Carlsbad, CA), and the gel was run following the manufacturer’s protocol. One lane containing Sharp prestained standards and the accompanying lane loaded with one-fourth of the sample were excised and stained using a silver nitrate protocol (QuickSilver; Pierce). Silver nitrate-stained lanes were realigned with the unstained gel section to create a template for excision. Unstained lanes were segmented into 14 equal sections and subjected to in-gel tryptic digestion at 57°C for 16 h following the procedure of Shevchenko et al. (67). One portion of the digested sample was mixed with an equal volume of a saturated solution of α-cyano-4-hydroxycinnamic acid (CHCA) acid in 0.1% trifluoroacetic acid (Pierce, Madison, WI) and spotted onto a stainless steel target plate with matrix-assisted laser desorption ionization–time of flight (MALDI-TOF) analysis on an AutoFlex III TOF mass spectrometer (Bruker, Billerica, MA) to determine the quality of digestion. The rest of the gel extract was lyophilized, and concentrated peptides were rehydrated in 15 μl of a solution of 0.1% formic acid and 5% liquid chromatography-mass spectrometry (LC-MS)-grade acetonitrile (ACN). The peptides were desalted using home-brew StageTips that involved loading 4 μl of peptides on a Dionex 3000 nano rapid-separation liquid chromatography (RSLC) series high-performance liquid chromatography (HPLC) system (Thermo Electron, USA) at the rate of 2 μl/min onto a precolumn packed with 5-μm YMC ODS-C18 beads (Waters, Milford, MA). Desalted peptides were passed through an analytical column containing Halo solid-core C18 particles with a pore size of 300 Å (Advanced Material Technology, Wilmington, DE, USA). Samples were eluted with a linear gradient of 95% buffer A (0.1% formic acid [Pierce], 5% acetonitrile [Honeywell], and 94.9% LC-MS-grade water) to 55% buffer B (90% ACN, 9.9% water, and 0.1% formic acid [FA]). Eluted sample from LC-MS was directed to the electrospray source of a linear ion trap mass spectrometer (LTQ/XL; Thermo Electron, USA). Tandem mass spectrometry (MS-MS) spectra were acquired, and the six most intense peaks from each spectrum were selected. The raw data set of peptides was then refined to a centroid list using Distiller (version 2.5; Matrix Science, Cambridge, UK), and matched to staphylococcal protein sequences in the UniProt database of 15 October 2015 using the MASCOT 2.5 database search engine (Matrix Science, Cambridge, UK). Spectral data sets were also processed and searched with the SpectrumMill proteomic workbench (Rev A.03.02.060; Agilent Technologies, Santa Clara, CA). A minimum peptide ion score cutoff of 7 was set. Alignments from both engines were merged and curated using Scaffold (v3.6.4; Proteome Software, Portland, OR). Scaffold software was used to rescore matches using the Protein Prophet algorithm. Scaffold results were restricted to a protein false-discovery rate of less than 1% and with protein confidence of more than 90%. Proteins with at least two unique peptides were chosen.

### Bioinformatic analysis and screening of the transposon mutant library.

The genome sequence of community-associated MRSA (CA-MRSA) USA300 strain FPR3757 was analyzed using web-based tools to identify proteins that contain a secretory signal or anchor to the cell wall or cell membrane with domains exposed outside the cell. USA300 strain FPR3757 is closest genetically in terms of sequenced strains. The presence of a signal peptide in the target open reading frame (ORF) was predicted using SignalP and PSORTb v.3.0p (68, 69). Similarly, localization on the cell wall and on the cell membrane with extended regions facing outside cytoplasm was predicted by PSORTb v.3.0 and TMHMM v.2.0 (68, 70). Proteins were classified with respect to their location as either extracellular, cell wall associated, membrane, cytoplasmic, or unknown as determined by SignalP, PSORTb v.3.0, and TMHMM v.2.0 (68–70). Proteins that contain secretory signal, anchor to the cell wall, and contain transmembrane helices with exposed N- or C-terminal domains were chosen for further analysis.

A transposon library containing mutations in the selected ORFs was analyzed for biofilm formation in the presence of heparin and compared with the wild-type strain. Briefly, selected mutants from the Nebraska Transposon Mutant Library (NTML) (20) were grown overnight in TSB from single colony on TSA plates. Overnight cultures were diluted 1:200 into biofilm medium in the wells of microtiter plates and were grown for 18 h at 37°C and 200 rpm. The biofilm assay was performed as discussed in the previous section (see “Microtiter plate-based biofilm assay” above).
