# Supplementary figures and images for: Heparin Mimics Extracellular DNA in Binding to Cell Surface-Localized Proteins and Promoting Staphylococcus aureus Biofilm Formation
Source: mSphere. 2017 Jun 21;2(3):e00135-17. doi: 10.1128/mSphere.00135-17 (PMC5480030; doi:10.1128/mSphere.00135-17)

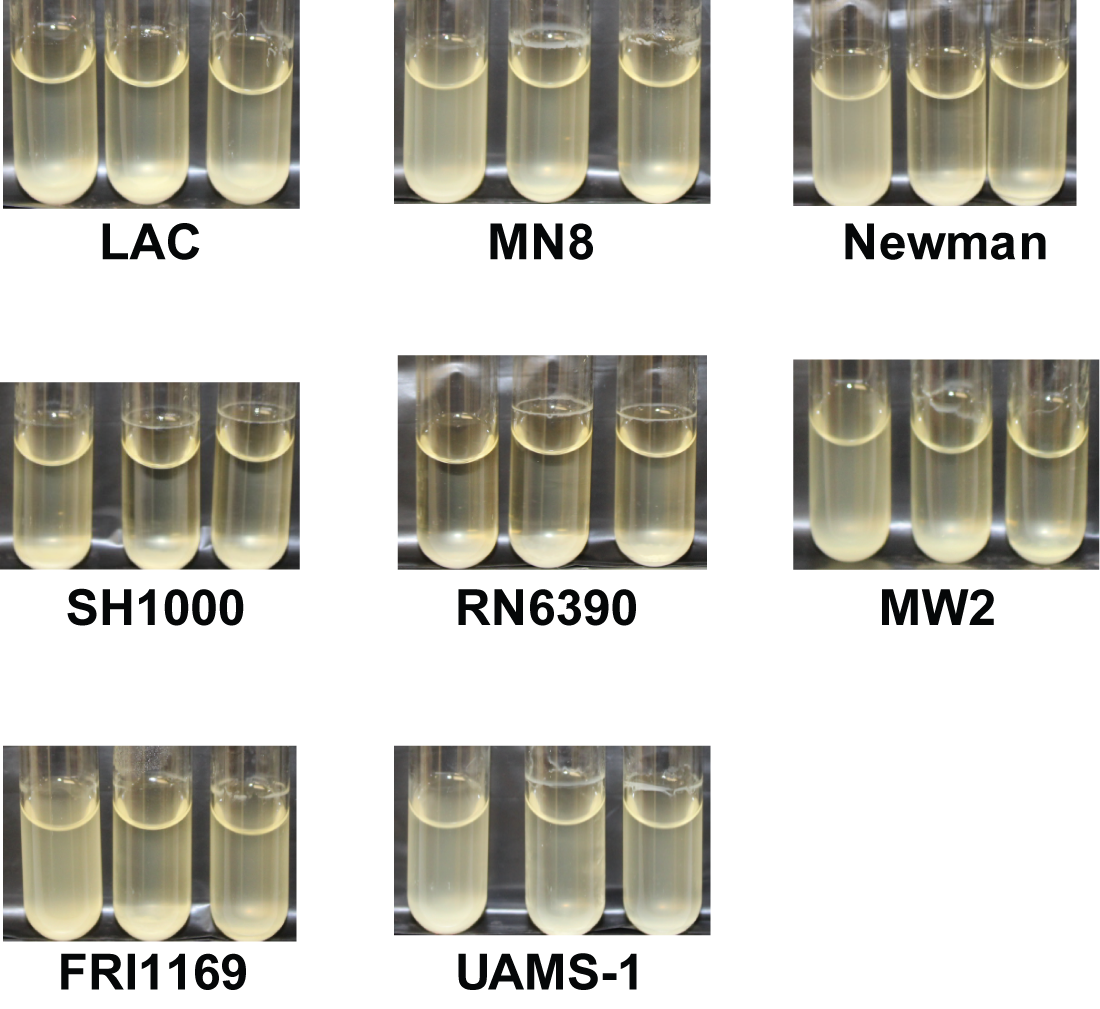

Supplement: FIG S1 [file sph003172307sf1.tif]

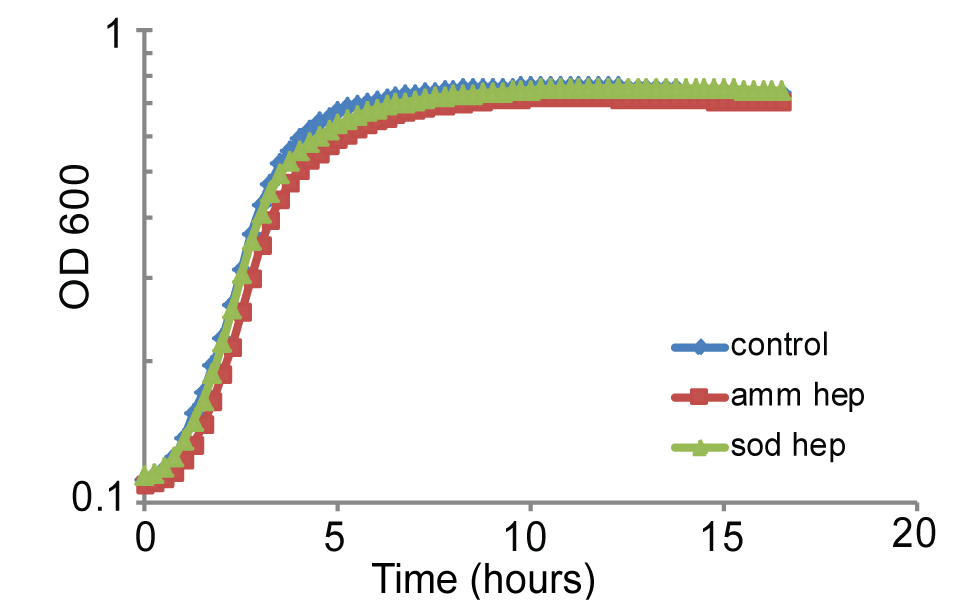

Supplement: FIG S2 [file sph003172307sf2.tif]
